# Regional muscle features and their association with knee extensors force production at a single joint angle

**DOI:** 10.1007/s00421-023-05237-w

**Published:** 2023-05-31

**Authors:** Andrea Monte, Martino V. Franchi

**Affiliations:** 1 Department of Neurosciences, Biomedicine and Movement Sciences, University of Verona, Verona, Italy; 2 Department of Biomedical Sciences, University of Padua, Via Marzolo 3, 35131 Padua, Italy; 3 CIR-MYO Myology Centre, University of Padua, Padua, Italy

**Keywords:** Muscle volume, PCSA, Muscle force, ACSA, Muscle strength, Muscle architecture

## Abstract

This study aimed (i) to investigate the role of regional characteristics of the knee extensors muscles (vastus lateralis: VL, vastus intermedius: VI and rectus femoris: RF) in determining maximum-voluntary force (MVF); and (ii) to understand which regional parameter of muscle structure would best predict MVF. Muscle architecture (e.g., pennation angle and fascicle length), muscle volume (Vol), anatomical (ACSA) and physiological cross-sectional-area (PCSA) were measured in the proximal (0–33% of the muscle length), middle (33–66% of the muscle length) and distal (66–100% of the muscle length) portions of each muscle in fifteen healthy males using ultrasound and Magnetic Resonance Imaging (MRI). Knee extensors force was calculated in isometric condition at a single knee joint angle of 90 degrees. Regional ACSA, Vol and PCSA were correlated with MVF production. Regional muscle geometry showed no significant correlations with MVF. Among regions, the middle portion of each muscle was largely correlated with MVF compared to all the other regions (distal and proximal). To understand which regional structural parameter best predicted MVF, a stepwise multiple linear regression was performed. This model showed a significant explanatory power (P < 0.001, R^2^ = 0.76, adjusted R^2^  = 0.71), including muscle Vol collected in the mid portions of VL and RF. Even if no significant differences were reported between Vol, PCSA and ACSA in determining MVF, our results showed that the RF and VL volume collected in the middle portion of the muscle length are strong determinants of MVF produced by the knee extensors at 90 degrees joint angle.

## Introduction

The capacity of a muscle to generate maximal force (i.e., as expressed by maximum voluntary isometric force production—MVF) depends on several parameters, such as muscle size (Narici et al. 1996; Blazevich et al. 2009), architecture (Lieber et al. 2017), muscle phenotype (Schiaffino and Reggiani 2011) and the level of muscle activation (Aagaard et al. 2002).

Regarding muscle size, anatomical cross-sectional area (ACSA) and muscle volume are two of the major determinants of muscles mechanical output (Balshaw et al. 2021; Maden-Wilkinson et al. 2021). Muscle adaptations in response to mechanical loading (Folland and Williams 2007), as well as degenerative processes following immobilization (Oates et al. 2010), unloading (Monti et al. 2021a; Franchi et al. 2022) and ageing (Narici et al. 2003, 2008), involve decrements in ACSA and muscle volume, linked to a diminished capability for force production. On one hand, an increase in pennation angle may facilitate the packing of more contractile material independently of changes in ACSA (Narici 1999). The effect of muscle arrangement is theoretically best reflected by its physiological cross-sectional area (PCSA), defined as the section that cuts all the fibers at the right angle (Narici 1999; Lieber et al. 2017), which most accurately reflect the maximum tetanic tension that can be generated by the muscle, and recently proposed as the best variable to use for force normalization (Lieber 2022). As suggested by Lieber (2022), the PCSA could be calculated by knowing muscle mass, muscle density and fiber length. Theoretically, if a given muscle would present a “cylindrical” shape, by dividing its volume by its length we would obtain a cylinder cross-sectional area. Of course, for pennate muscle, fiber length does not equal to muscle length. Therefore, PCSA would not actually represent any definable anatomical area; rather, it would represent a theoretical area that would be occupied by a cylinder with a length equal to that of the fibers. Since muscle size and fascicle length could exhibit regional variation along femur length (Blazevich et al. 2006), then PCSA could also differ along femur length.

Therefore, a rigorous assessment of the regional structural properties of a given muscle (volume, ACSA, PCSA and muscle geometry) could provide important information on its capacity to generate torque.

Several previous studies have highlighted the effects of structural properties on muscle force generation. For example, positive relationships were observed between knee extension MVF and quadriceps ACSA (measured at 50% of the femur length) in both men (r = 0.71) and women (r = 0.76) (Jones et al. 1989). The role of muscle volume and PCSA of the vastus lateralis was instead evaluated by Blazevich et al. (2009). The authors showed that muscle volume was typically the best predictor of knee extensor moment during fixed-end and dynamic contractions.

Two recent studies tried to identify the most important structural parameters in determining muscle MVF. Maden-Wilkinson and colleagues (Maden-Wilkinson et al. 2020) highlighted that the most important parameters in determining muscle strength are muscle volume and its PCSA. In particular, the authors showed that the differences in the quadriceps muscle force between trained and untrained populations are explained by the differences in muscle volume and PCSA, rather than muscle geometry (fascicle length and pennation angle). More recently, Balshaw et al. (2021) showed that the statistical comparisons of correlation coefficient between the association of PCSA, ACSA or VOL with MVC revealed no difference among dependent variables. These studies suggested that muscle strength derives from a complex interaction between structural parameters (e.g., PCSA, ACSA or VOL), rather than by just one determinant or by muscle architectural arrangement. However, as previously observed, muscle architecture of the knee extensors differs along the femur length (Blazevich et al. 2006; O’Brien et al. 2010; Wakahara et al. 2013). Since the amount of muscle mass and the fascicle length could exhibit regional variation along femur length (for the case of VL muscle), the three main parameters (volume, ACSA, and mostly PCSA) could also differ as a function of it. In this regard, Trezise et al. (2016) studied the correlations between the structural parameters of quadriceps muscles (assessed at different percentages of femur length) and MVF, showing that the proximal region (30% of the thigh length) of the knee extensors was more correlated with MVF than the other regions (middle and distal). Further, using a statistical model, the authors concluded that the most important parameter in determining MVF in the quadriceps muscles was the proximal ACSA, rather than the traditionally used mid-section ACSA.

Taken together, these studies provided important indications: (1) muscle volume, ACSA and PCSA seem to be the structural parameters which are better associated to maximal torque production, but; (2) regional differences could play an important role when investigating the maximal force capacity of a muscle, and such difference are often neglected. In fact, the in-vivo calculation of muscle PCSA considers fascicle length value only calculated in the middle region of the muscle. However, different training strategies have also been shown to affect muscle geometrical arrangement in a regional manner (Seger et al. 1998; Franchi et al. 2014), and therefore the evaluation of PCSA in different sub-regions (considering different regional portions of muscle as a smaller cylinders/truncated cones) could potentially enhance the meaning of PCSA per se, as this could be helpful when further analyzing regional effects of training, disuse, and disease on muscle morphological and functional characteristics.

Therefore, the present study aimed to better understand the effects of the most important structural parameters and their regional characteristics in determining knee extensors maximum voluntary force. Furthermore, we investigated which structural parameter (and region) would best predict muscle force generation. Due to the highest amount of muscle mass situate in the middle region, we expected to find a predominant role of the middle regional in determining MVF. Finally, based on the previous literature, we did not expect a superior effect of muscle volume, PCSA or ACSA in affecting MVF.

## Material and methods

### Participants

Fifteen healthy trained males (age: 35 ± 3 years; body mass: 78 ± 6.8 kg; height: 1.76 ± 0.08 m; number of endurance training sessions per week: 3 ± 1) participated in this study. All participants were moderately active and were involved in recreational sport activities (mainly running or cycling). The study agreed with the Declaration of Helsinki for the study on human participants. The local ethical committee approved the experimental protocol, and all participants gave their written informed consent.

### Experimental design

The volunteers attended three different experimental sessions (24 h in between each visit). In the first visit, an MRI scan of the right thigh was performed. In the second visit, muscle architectural parameters (pennation angle and fascicle length) of the knee extensor muscles (Vastus Lateralis: VL, rectus Femoris: RF, Vastus Intermedius: VI and Vastus Medialis: VM) were measured using in B-Mode ultrasonography. Ultrasound images were recorded at three different locations (i.e., proximal, middle, and distal) along the length of each muscle using a linear array probe of 45 mm. Two images were collected in series to obtain a total field of view of 90 mm (for more details see the next section). After that, an isokinetic dynamometer was used to perform 4 maximal isometric voluntary contractions of the knee-extensor muscles. During the third visit, the same procedures used in the second visit were repeated.

### Protocol

#### MRI scan

After 1 h of supine rest to control for the influence of postural related fluid shifts on muscle size, MRI scans were obtained for each participant. Participants were supine, and their feet attached to a non-metallic support to avoid joint displacement and scan angle and to minimize compression of the legs against each other and the MRI gurney. Imaging was completed in a 1.5 Tesla Magnetom Symphony (Siemens, Erlangen) to determine the volume (Vol) and ACSA of the rectus femoris, vastus lateralis and vastus intermedius of the right limb. A coronal scout scan [repetition time/echo time (TR/TE) 5300/14 ms, field of view 48 cm, 256 × 160 matrix] of 5 slices of 5 cm thick with 5-mm spacing was completed to establish orientation of the femur. After the scout scan, interleaved transaxial images of 1 cm thick (TR/TE 633/20 ms, field of view 274 × 480 mm, 256 × 256 matrix) were taken from the top of the greater trochanter of the femur to the articular surface of the tibia.

#### Ultrasound measurements

For each muscle (VL, VI and RF), fascicle length and pennation angle were recorded using two-dimensional B-mode ultrasonography (HeathCare S7 pro, GE) using a 9 MHz 45 mm linear-array probe. After 10 min of resting supine, scans were acquired for the different regions of interests. First, the origin and the insertion of each muscle were identified by means of the ultrasound and highlighted with a skin mark. After that, each muscle was divided into three different sections: proximal (0–33% of the muscle length), middle (33–66% of the muscle length) and distal (67–100% of the muscle length). Then, the middle point of each section [16% (proximal), 50% (middle) and 83% (distal) of each muscle length] were identified by means of a skin marker (iron wire) to see the marker location with the ultrasound. Finally, ultrasound images were taken after and before each surgical marker to obtain a field of view of ~ 9 cm for each portion. Similar procedures were used by several authors (Noorkoiv et al. 2010; Ando et al. 2016). During these procedures, a rolled towel was placed underneath the knee joint to remove compression of the muscles. These procedures were repeated three times. All measurements were manually traced using ImageJ software (1.41o, National Institute of Health, USA).

#### Dynamometric measurement

For the measurement of knee extensors maximum isometric torque, the participants were secured on a dynamometer (Byodex NORM, USA), fixed with a trunk and pelvic strap and the arms positioned crossed in front of the chest. Hip and knee joint angle were set at 85° and 90°, respectively (0° refers to supine position).

The real knee angle and the patellar tendon moment arm were measured using two-dimensional kinematics as utilized by Monte et al. (2020). Briefly, the knee joint angles and the patellar tendon moment arm were obtained by knowing the position of five markers: greater trochanter; lower portion of the patella (patellar tendon origin); upper anterior surface of the tibia (patellar tendon insertion); mid-tibiofemoral gap (considered to represent the tibiofemoral contact point as the knee center of rotation) and lateral malleolus. The marker positions were recorded by means of smartphone at 120 Hz and analysed with a video processing software (Tracker v4.0).

### Data analysis

#### Ultrasound

**Fig. 1:**
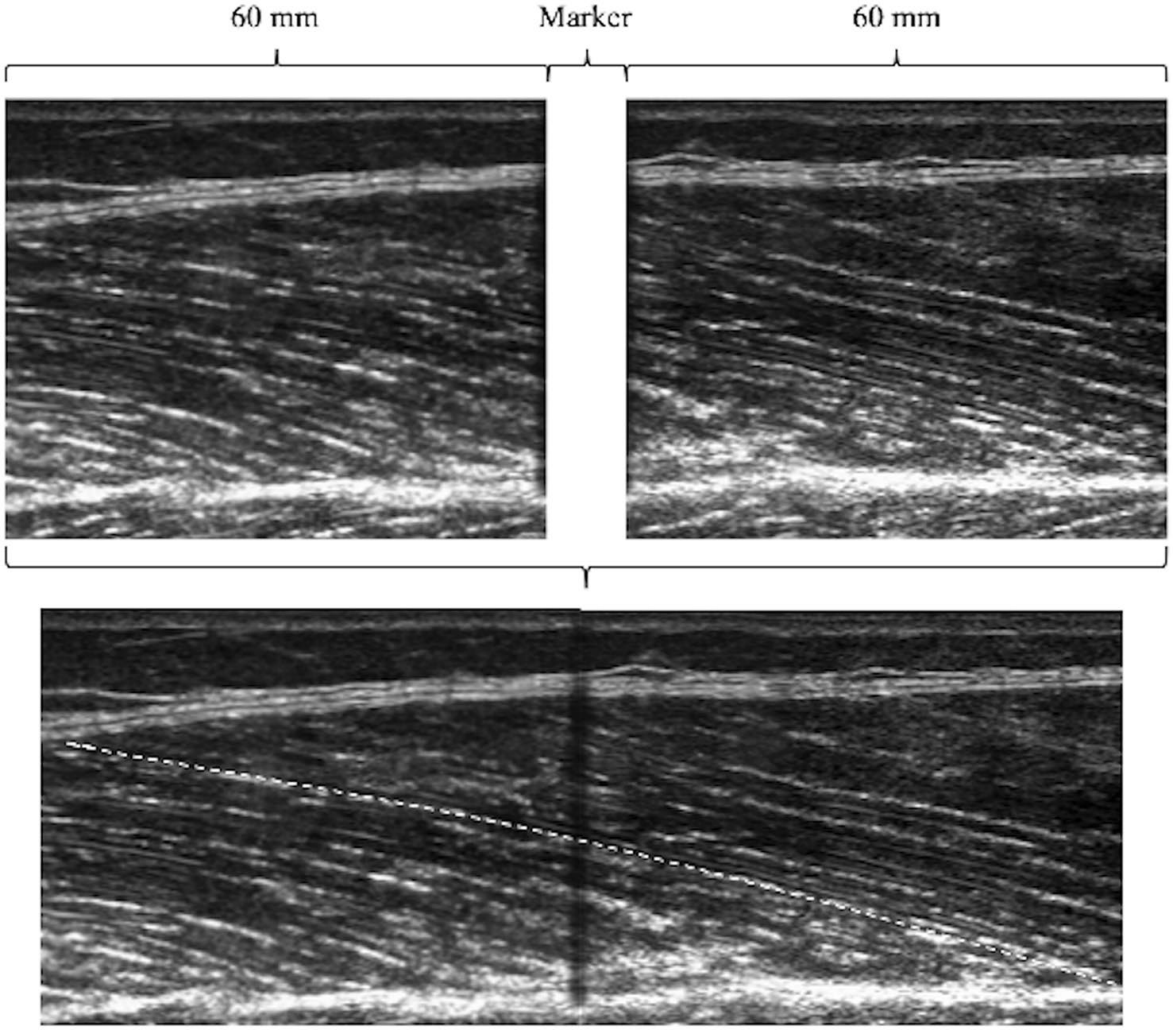
Example of the image stitching procedure. Two different images have been combined in one single shot, based on the reference point

For the geometrical arrangement, the two single images (45 mm of view) that compose a reference point were matched to obtain an extended field of view of 9 cm (see Fig. 1).

This procedure was visually performed using a customized Matlab program by means of the imaging process toolbox (Matalb v.2016). At the end of the imaging matching process, 9 images for each muscle (VL, RF and VI) were obtained: three for each region (proximal, middle, and distal). The data of VM were excluded from the data analysis due to its complex architectural arrangement, difficult to capture without compromising the quality of the scans. As poor image quality was obtained, which prevent us to fully trust our measurements, we decided to not report any data for VM muscle.

**Fig. 2:**
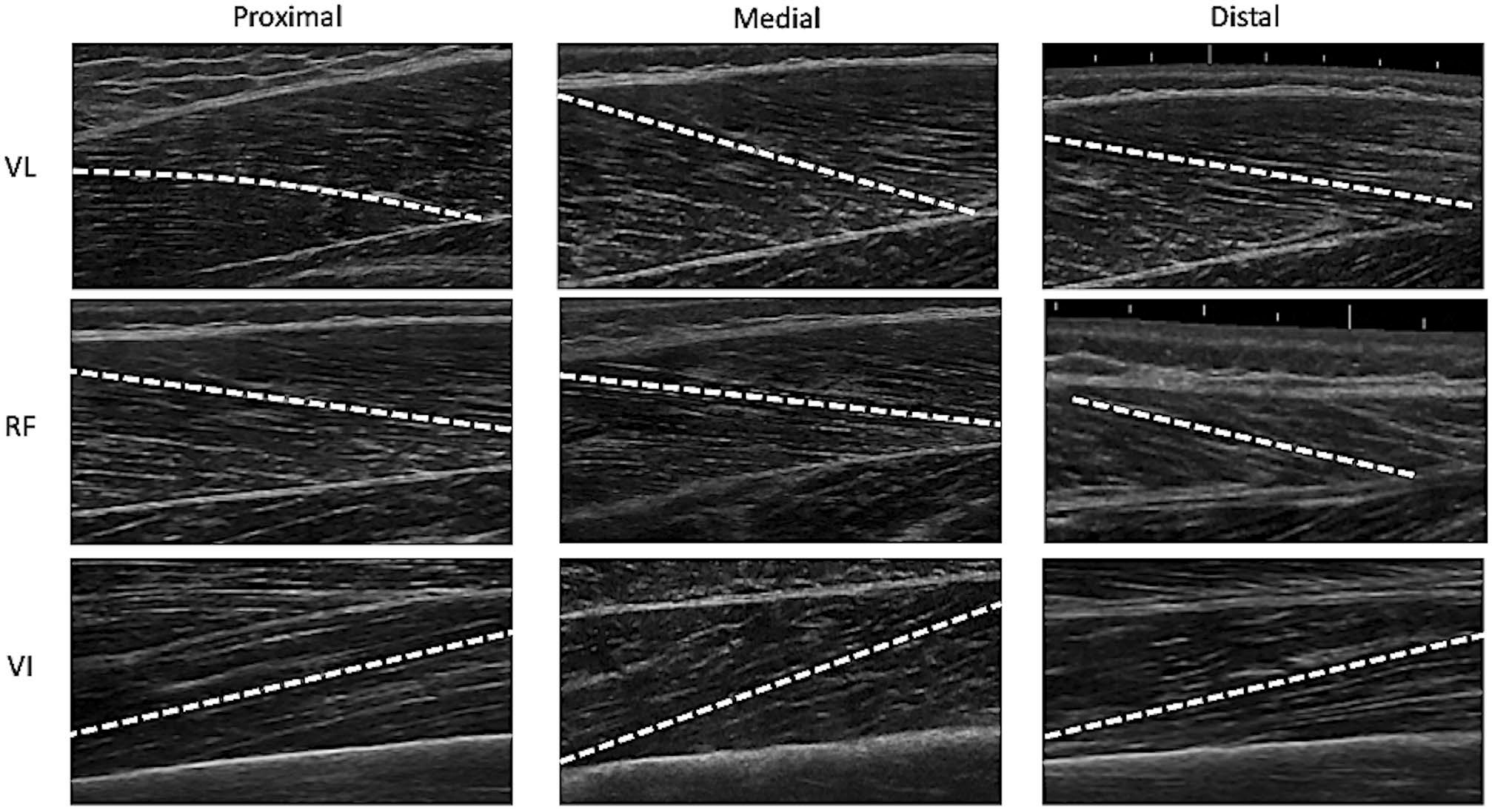
Representative ultrasound images for vastus lateralis (VL), rectus femoris (RF) and vastus intermedius (VI) in all the investigated regions

For each ultrasound image, the angle between the echo of the deep aponeurosis of the muscle and the interspaces among the fascicles of the muscles was taken as pennation angle. Finally, fascicle length was manually traced form the deep to the superficial aponeurosis (Fig. 2). All images were analyzed with ImageJ by the same operator.

To check the reliability of the analyses, we tested the interclass correlation coefficient and the coefficient of variation. Fascicle length was used for this analysis because it represents the parameters more affected by this procedure. The ICC range were: 0.90–0.93, 0.92–0.97 and 0.91–0.94 at the proximal, middle, and distal point (means among muscles), respectively. The typical CV ranges were: 3.5–4.1%, 2.3–3.7% and 2.8–3.8% at the proximal, middle, and distal point (means among muscles), respectively.

#### MRI

After each measurement, all images were analyzed with OsiriX (version 3.7.1 32 bit) using manual planimetry tool. The ACSA of each of the three heads of the quadriceps femoris was manually outlined on each slice of thigh. The regional muscle volume and ACSA were obtained as described for the ultrasound images (0–33, 34–66, 67–100% of each muscle length for the distal, middle, and proximal region, respectively). The mean values obtained for each region was utilized for further analysis. The same investigator carried out all the measurements. Proximal, middle, and distal physiological cross-sectional area (PCSA) was calculated based on the ratio between the regional volume and the corresponding fascicle length.

Finally, the total proximal, distal, and middle ACSA, PCSA and volume were calculated as the sum of regional characteristic of the three investigated muscles (e.g., _TOT_ACSA_prox_ = VL ACSA_prox_ + VI ACSA_prox_ + RF ACSA_prox_).

#### Dynamometry

The total moment generated by the knee extensor was corrected for the gravitational moment effects (determined during a passive joint rotation driven by the dynamometer).

For the knee extensor, the lever arm was measured as the perpendicular distance from the tendon’s line of action to the center of rotation of the knee as used by Monte et al. (2020). Finally, by knowing the maximum isometric torque generated by the knee extensors and the moment arm, maximum quadriceps isometric force (MVF) was calculated.

### Statistical analysis

Normality distribution of the data was assessed using the Saphiro-Wilk test. Since all variables resulted normally distributed a one-way (factor: region) ANOVA was used to check the geometrical differences (pennation angle, fascicle length and ACSA) between muscle region (proximal, middle, and distal) in all the investigated muscles. *N* was equal to fifteen for all the investigated variables. No subjects were excluded. Pearson’s correlations coefficient was used to check the possible relationships between MVF and all the investigate variables. If two region of the same muscle showed significant correlations with MVF, equality of correlation coefficient (Hotelling’s statistics) was performed to check which region best predict the MVF. Hotelling’s test was also used to determine the most important MVF predictors among the total regional parameters. Finally, a stepwise multiple linear regression models were used to identify the most important individual muscular parameters in determining MVF.

These statistical analyses were performed with R (v.31) and the level of significance was set to P < 0.05.

## Results

Maximum voluntary torque and force were 279 ± 21 Nm and 6488 ± 340 N, while the patellar tendon moment arm was 4.43 ± 0.25 cm. Total volume (from 0 to 100% of muscle length) of VL, RF and VI was respectively 636.4 ± 34, 280.6.4 ± 26 and 553.4 ± 29 cm^3^, while the total PCSA was, respectively, 63.6 ± 5.2, 40.1 ± 3.2, 41.6 ± 3.4 cm^2^.

The ACSA, volume and PCSA of all investigated muscles were significantly affected by the regional portion (P < 0.001 in all the investigated variables) (Table 1). In all the investigated muscles, middle portion showed the largest ACSA, volume and PCSA. Pennation angle was also affected by the regional portion, but only for RF (P < 0.01) and VI (P < 0.001). Fascicle length showed significant effect of the regional side only in RF (P < 0.05). Post-hoc comparisons are reported in Table 1.

**Table 1:** Muscle structural variables for each portion of muscle length (distal, middle and proximal)

	Distal (0–33%)	Middle (34–66%)	Proximal (67–100%)
VL			
ACSA (cm^2^)	13.1 ± 0.6^+#^	28.2 ± 3.3*^#^	17.5 ± 2.8*^+^
Fl (cm)	9.9 ± 0.9	10.0 ± 0.9	10.1 ± 0.9
Pen (deg)	15.8 ± 0.8	15.5 ± 0.9	15.2 ± 0.9
Vol (cm^3^)	171.8 ± 19^+#^	337.0 ± 26*^#^	127.2 ± 10*^+^
PCSA (cm^2^)	17.3 ± 2^+#^	33.7 ± 6*^#^	13.1 ± 5*^+^
RF			
ACSA (cm^2^)	4.7 ± 0.5^+#^	7.3 ± 0.8*^#^	3.6 ± 0.5*^+^
Fl (cm)	8.5 ± 0.9^+#^	6.9 ± 0.8*	6.6 ± 0.9*
Pen (°)	16.3 ± 0.8^+#^	22.3 ± 0.8*	23.3 ± 0.9*
Vol (cm^3^)	62.0 ± 3^+#^	141.4 ± 11*^#^	77.2 ± 8*^+^
PCSA (cm^2^)	7.8 ± 1^+#^	18.9 ± 2*^#^	10.0 ± 1*^+^
VI			
ACSA (cm^2^)	12.9 ± 0.8^+#^	23.5 ± 2*^#^	15.6 ± 0.9*^+^
Fl (cm)	6.8 ± 0.8	7.0 ± 0.8	7.2 ± 0.8
Pen (°)	10.2 ± 0.8^+#^	13.1 ± 0.8*^#^	16.0 ± 0.9*^+^
Vol (cm^3^)	124.4 ± 10^+#^	283.8 ± 22*^#^	145.3 ± 13*^+^
PCSA (cm^2^)	17.5 ± 4^+#^	40.3 ± 7*^#^	20.7 ± 6*^+^
Total			
ACSA (cm^2^)	30.7 ± 3.4^+#^	59.0 ± 4.3*^#^	36.7 ± 3.1*^+^
Vol (cm^3^)	358.2 ± 28^+#^	762.2 ± 37*^#^	349.7 ± 38*^+^
PCSA (cm^2^)	42.6 ± 6.8^+^	92.9 ± 9.2*^#^	43 ± 7.1^+^

Total ACSA, Vol and PCSA refers to the sum of all the investigated muscles in that region

Proximal: (0–33% of the muscle length, middle: 33–66% of the muscle length and distal: 67–100% of the muscle length

*ACSA* anatomical cross-sectional area, *Fl* fascicle length, *Pen* pennation angle, *Vol* volume, *PCSA* physiological cross-sectional area

*Significant difference compared with distal

^+^Significant difference compared with middle

^#^Significant difference compared with proximal

The total ACSA, volume and PCSA were significantly affected by the regional portion (P < 0.001 in all the investigated variables). The middle portion showed the highest ACSA, PCSA and volume, compared to the other regions.

### Correlations between MVC and structural parameters

Among the structural variables, only the ACSA, volume and PCSA showed positive significant correlations with MVF (see Table 2). On the contrary, pennation angle and fascicle length were no significant correlated with MVF. The highest correlation coefficient was observed for the volume of the VL taken in the middle portion of muscle length (see Table 2). Finally, patellar tendon moment arm was also significantly correlated with MVF.

**Table 2:** Correlations coefficients between individual structural properties and MVF

	Distal (0–33%)	Middle (34–66%)	Proximal (67–100%)	Hotelling’s test
VL				
ACSA (cm^2^)	0.37	0.59*	0.49	
Fl (cm)	0.13	0.15	0.33	
Pen (°)	0.19	0.46	0.37	
Vol (cm^3^)	0.55*	0.75***	0.55*	Mid > Prox = Dist
PCSA (cm^2^)	0.48	0.68**	0.55*	Mid > Prox
RF				
ACSA (cm^2^)	0.44	0.51*	0.49	
Fl (cm)	0.16	0.28	0.13	
Pen (°)	0.11	0.04	− 0.08	
Vol (cm^3^)	0.51*	0.62*	0.53*	NS
PCSA (cm^2^)	0.44	0.56*	0.49	
VI				
ACSA (cm^2^)	0.43	0.50*	0.45	
Fl (cm)	0.16	0.15	0.03	
Pen (°)	0.17	0.22	0.25	
Vol (cm^3^)	0.41	0.52*	0.40	
PCSA (cm^2^)	0.43	0.51*	0.48	
Total				
ACSA (cm^2^)	0.49	0.70*	0.47	
Vol (cm^3^)	0.56*	0.80***	0.55*	Mid > Prox = Dist
PCSA (cm^2^)	0.48	0.78**	0.49	
Moment arm		− 0.52*		

*ACSA* anatomical cross-sectional area, *Fl* fascicle length, *Pen* pennation angle, *Vol* volume, *PCSA* physiological cross-sectional area

*P < 0.05; **P < 0.01; ***P < 0.001

In VL, Hotelling’s test was used to compare the differences between regional muscle volume and PCSA in determining MVF, showing that in both cases the middle region better describes MVF. Hotelling’s test was also used to evaluate the difference in RF muscle volume distribution, showing no significant different among region in determining MVF. In the middle region of each muscle, ACSA, PCSA and muscle volume were significantly correlated with MVF. Therefore, an additional Hotelling’s test was run to identify the most important parameter in determining MVF. The results did not show any significant difference in determining MVF, suggesting that the ACSA, PCSA and muscle volume collected in the middle region of each muscles play similar role in affecting MVF.

When the regional muscle’s characteristics are considered together (sum of the three muscles), ACSA and PCSA collected in the middle portion were significantly correlated with MVF. On the contrary, proximal and distal value of total ACSA and PCSA were not correlated with MVF. To note, total regional muscle volume was positively correlated with MVF in all the investigated regions (Table 2). Among regions, Hotelling’s test showed that the volume of the middle portion best predicts MVF, compared to the other regions. Since the total ACSA, PCSA and volume collected in the middle region were positively correlated with MVF, an Hotelling’s test was used to identify the main predictor. No significant differences were reported among total ACSA, PCSA and volume collected in the middle region.

To understand which kind of regional structural parameters would best predict the maximum voluntary force, a stepwise multiple linear regression was performed. The significant regional parameters reported in Table 2 were included in the model. Due to the collinearity among variables, each parameter was standardized (e.g., by subtracting the mean) before it was used in the model.

The resultant model was:

$$\text{MVF} = -{195} \, \text{+} \, \text{0.18} \, {VOL}_{VLmid} \, \text{+} \, \text{0.44 }{VOL}_{RFmid}$$

This model resulted in a significant explanatory power (P < 0.001, R^2^ = 0.76, adjusted R^2^ = 0.71). The standardize beta coefficients were: 0.471 (P = 0.004) and 0.331 (P = 0.013) for VOL_VLmid_ and, VOL_RFmid_, respectively.

## Discussion

Our results showed that the middle region of each investigated muscle was more correlated to maximum isometric force compared to the other regions when measured at a single joint angle of 90 degrees. Despite muscle architecture features showed no significant correlation with MVF (although a strong tendency (R^2^ = 0.46) was found for pennation angle in the mid-portion of VL), ACSA, PCSA, and volume collected in the middle portion of each muscle were positively correlated with MVF. Conversely, the same measures in distal and proximal regions showed low or no correlation with MVF. This was also particularly evident when considering all muscles together (total ACSA, PCSA and volume). Indeed, among all parameters, our multiple linear regression model showed that the VL and RF muscle volume collected in the middle region play a primary role in determining MVF.

It must be noted that, for the present study, we chose a knee angle of 90 degrees as this angle has been adopted in many previous studies, both during maximum and explosive contractions, in studies investigating both hypertrophic and atrophic states (Blazevich et al. 2006, 2009; Tillin et al. 2010; Monti et al. 2021a, b; Sarto et al. 2022). In addition, we decided to not investigate vastus medialis due to its complex geometrical arrangement (and subsequent difficulty to be scanned with sufficient reliability), as described in the methods section.

### Determinants of maximum isometric force

Muscle architecture parameters were not correlated to MVF (see Table 2) compared to the measures of muscle size (i.e., VOL, ACSA) or PCSA. Theoretically, although changes in fascicle length could affect the in-vivo angle-torque relationship (Hinks and Franchi 2022), fascicle length could be considered a determinant of muscles contraction velocity rather than influencing pure force production (Lieber et al. 2017). Indeed, the larger the number of in-series sarcomeres, the higher should be the maximum contraction velocity (Lieber et al. 2017). On the contrary, pennation angle could play an important role in determining maximum isometric force, being more reflective of muscle radial hypertrophy (Lieber et al. 2017). (Trezise et al. 2016), showed that the values of vastus lateralis pennation angle collected in the middle portion of the muscle length were positively correlated with knee extensors maximum isometric force (r = 0.39), suggesting that pennation angle played an important role in determining quadriceps muscle torque. Our data of VL pennation angle agree with those reported by Trezise and co-workers, although the correlation with MVF was 0.46 (P = 0.055). Future studies with larger sample size should check this result. Conversely, in agreement with Trezise et al. 2016), pennation angle of the other muscles seem to not be of primary importance in determining MVF. However, it must be noted that a recent article has shown that in-vivo muscle architecture values measured at rest may be less representative of the ones measured at the optimal joint angle for force production (Werkhausen et al. 2023), thus being a potential limitation of the present study and something to take in consideration for future investigations.

Our data showed that ACSA was a good predictor of knee isometric extensor force at 90 deg joint angle. Previous investigations showed that the ACSA was correlated with joint force in several tasks (e.g., dynamic and isometric) for different muscle groups (e.g., knee extensors, knee flexors and plantar flexors) (Fukunaga et al. 1997; Maden-Wilkinson et al. 2020). Our results suggest that ACSA could be a good predictor of muscle strength when calculated in the middle region of the muscle length, probably due to the higher muscle mass enclosed in this portion (Table 1). Nevertheless, as regional hypertrophy has been previously observed especially after eccentric training (Franchi et al. 2014; Seger et al. 1998), we question whether mid portion ACSA would still represent the best predictor of MVF after chronic exposure to such training regimes. However, it must be pointed out that Trezise et al. (2016) showed a superior role of the proximal ACSA in determining MVF, compared to the middle one. This discrepancy can be explained by the different joint angles utilized during the MVF evaluation. Treizise and colleagues utilized more open knee joint angles compared to the one adopted in the current study.

Interestingly, PCSA, which is often considered the best predictor of muscle strength (Balshaw et al. 2021; Lieber 2022), was not the strongest predictor. Theoretically, the simultaneous inclusion of ACSA and fascicle geometrical arrangement would result in a prominent role of PCSA in determining MVF. Indeed, greater fascicle angulation allows more contractile tissue to attach to a given area (Narici 1999; Lieber et al. 2017), which would increase the possibility to generate more contractile force, and of course PCSA. However, in accordance with previous studies (Blazevich et al. 2009; Maden-Wilkinson et al. 2020; Balshaw et al. 2021), our data showed that the PCSA was not the primary determinant of MVF, possibly because fascicle lengths values used in the PCSA calculation have been obtained at rest with the leg placed in extended position (Werkhausen et al. 2023). Thus, muscle volume showed the largest determination coefficients, albeit not statistically different compared to ACSA and PCSA. Overall, the differences between these three indices of muscle size in predicting strength appear relatively subtle. Our data provide further insight about the role of PCSA. Indeed, we showed that the regional repartitioning of PCSA did not change its prediction capacity, remaining one of the predictors but not the best one. To note, our data showed that the PCSA is more correlated with MVF when collected in the middle region, probably since muscle volume is the highest in the same region.

Muscle volume appeared to be the strongest predictor of MVF, although it was not statistically different than ACSA or PCSA, as similarly reported in previous studies (Blazevich et al. 2009; Trezise et al. 2016; Balshaw et al. 2021). In agreement with recent studies (Maden-Wilkinson et al. 2020; Balshaw et al. 2021), it appears that the contemporary assessment of muscle architecture would offer no advantage over indices of muscle size; however, the fact that muscle architecture and ACSA differently changes along the whole muscle length during resistance training interventions still warrants further investigations, as it could affect force production at different joint angles (at shorter or longer muscle lengths). However, although muscle volume is a property of the entire muscle, it is well documented that training strategy could differently affect muscle regions, increasing (for example) the amount of ACSA in one specific region, leading to an increase in muscle volume in region-specific manner. Noteworthy, our data pointed out that the muscle volume collected in the middle region could be particularly involved in determining MVF at 90 degrees joint angle. Our linear regression model showed that the largest predictor of MVF was the muscle volume collected in VL and RF middle region. These results reinforce the idea that muscle volume could be considered a good predictor of MVF.

### Study limitations and further considerations

Muscle adaptations to training are specific to the nature of the training task undertaken. As an example, specific muscle adaptations can be induced using different joint angles (Noorkõiv et al. 2014, 2015) or distinct contraction types (Franchi et al. 2017). However, in this study, we utilized a single knee joint angle. This could represent a current limitation of our study, as we could assume that different results would be observed if different angles will be tested. We also speculate that adaptations in muscle architecture could play a more important role, as there is some evidence that, for example, longitudinal muscle growth (i.e., potentially reflected by an increase in fascicle length) is accompanied by a broadening of the plateau region of a muscle’s’ angle-torque relationship, thought to result in an optimization of force production at more joint angles (Kubo et al. 2006; Akagi and Hinks 2020). Considering that there is a consistent amount of literature showing changes in muscle architecture (Ema et al. 2016), also region-specific (Benford et al. 2021), we can speculate that such strategies of regional muscle remodeling would in turn influence muscle function at different joint angles, thus playing a role in determining MVF. Further studies are warranted to test such hypothesis.

We also acknowledge that our data have been collected at rest and with the leg placed in extended position: this could represent a limitation, as knee extensors MVF seems more related to architectural parameters collected closer to the optimal angle for force production (Werkhausen et al. 2023).

We acknowledge that the joint angle used during MVCs does not correspond to the joint angle utilized during the ultrasound imaging and MRI scans (supine position = 0 degrees). It is assumed that changes in joint angle would change result in the regional characteristics of the muscle due to changes in the length of the muscle fibers.

As reported in several studies, we used muscle architecture at rest as predictors of MVF. Future studies should consider using dynamic ultrasound analysis to better represent the determinants of MVF during contraction. Furthermore, data of VM were excluded from the data analysis due to its complex architectural arrangement. The absence of VM in our linear regression model could partially explain a part of variance which is not explained by the other muscles.

Lastly, the sample size adopted in this study could be small to detect small differences among parameters in determining MVF. Future studies with larger sample sizes could provide further insight into the role of regional muscle variation in determining muscle force.

## Conclusion

This study provided new evidence about the role of knee extensors regional characteristics in determining maximum isometric force. Muscle volume, PCSA and ACSA (and not muscle architecture per se) of the middle region of the investigated muscles plays an important role in determining in-vivo muscle force when produced at a 90 degrees knee angle, suggesting that this site may be a functionally relevant location for determining force contraction capacity for this specific joint angle. Among the others, even if the ACSA, PCSA and muscle volume can be considered good predictors of maximum quadriceps isometric force, the vastus lateralis and rectus femoris muscle volume collected in the middle region were the only ones included in the regression model.

## Data Availability

Data is available upon request.
